# Germline-Competent Mouse-Induced Pluripotent Stem Cell Lines Generated on Human Fibroblasts without Exogenous Leukemia Inhibitory Factor

**DOI:** 10.1371/journal.pone.0006724

**Published:** 2009-08-21

**Authors:** Chunliang Li, Hongyao Yu, Yu Ma, Guilai Shi, Jing Jiang, Junjie Gu, Ying Yang, Shibo Jin, Zhe Wei, Hua Jiang, Jinsong Li, Ying Jin

**Affiliations:** 1 Key Laboratory of Stem Cell Biology, Institute of Health Sciences, Shanghai Institutes for Biological Sciences, Chinese Academy of Sciences/Shanghai Jiao Tong University School of Medicine, Shanghai, China; 2 Shanghai Stem Cell Institute, Shanghai Jiao Tong University School of Medicine, Shanghai, China; 3 Laboratory of Molecular Cell Biology, Institute of Biochemistry and Cell Biology, Shanghai Institute for Biological Sciences, Chinese Academy of Sciences, Shanghai, China; 4 Key Laboratory of Cell Differentiation and Apoptosis of Chinese Ministry of Education, Shanghai Jiao Tong University School of Medicine, Shanghai, China; 5 Graduate School of Chinese Academy of Sciences, Beijing, China; KU Leuven, Belgium

## Abstract

Induced pluripotent stem (iPS) cells have attracted enormous attention due to their vast potential in regenerative medicine, pharmaceutical screening and basic research. Most prior established iPS cell lines were derived and maintained on mouse embryonic fibroblast (MEF) cells supplemented with exogenous leukemia inhibitory factor (LIF). Drawbacks of MEF cells impede optimization as well as dissection of reprogramming events and limit the usage of iPS cell derivatives in therapeutic applications. In this study, we develop a reproducible protocol for efficient reprogramming mouse neural progenitor cells (NPCs) on human foreskin fibroblast (HFF) cells via retroviral transfer of human transcriptional factors OCT4/SOX2/KLF4/C-MYC. Two independent iPS cell lines are derived without exogenous LIF. They display typical undifferentiated morphology and express pluripotency markers Oct4 and Sox2. Transgenes are inactivated and the endogenous *Oct4* promoter is completely demethylated in the established iPS cell lines, indicating a fully reprogrammed state. Moreover, the iPS cells can spontaneously differentiate or be induced into various cell types of three embryonic germ layers *in vitro* and *in vivo* when they are injected into immunodeficient mice for teratoma formation. Importantly, iPS cells extensively integrate with various host tissues and contribute to the germline when injected into the blastocysts. Interestingly, these two iPS cell lines, while both pluripotent, exhibit distinctive differentiation tendencies towards different lineages. Taken together, the data describe the first genuine mouse iPS cell lines generated on human feeder cells without exogenous LIF, providing a reliable tool for understanding the molecular mechanisms of nuclear reprogramming.

## Introduction

The generation of induced pluripotent stem (iPS) cells from mouse embryonic and adult fibroblasts by retroviral introduction of transcription factors (Oct4, Sox2, Klf4 and c-Myc) was first reported by Yamanaka and his colleagues in 2006 [1]. In the following year, the direct reprogramming of human somatic cells was accomplished [2], [3]. The enormous potential of iPS cells in therapeutic applications, pharmaceutical screening, *in vitro* disease modeling and molecular dissection of pluripotency has attracted considerable attention from the entire range of life sciences and has led to extremely rapid progress in this field. The iPS cells were initially obtained via drug selection [1]. However, it was quickly found out that they could be identified based solely upon morphological criteria [4], [5]. Soon, a drug-inducible reprogramming system was developed for multiple mouse cells [6]. Recently, the system has been further refined to allow removal of integrated transgenes from the host cell genome after reprogramming [7]. Meanwhile, gene delivery systems other than retro- or lenti- viral ones, such as repeated transfection with plasmids and use of a non-integration virus, have been successfully applied in generation of iPS cells [8], [9]. Moreover, various somatic cell types, including mouse liver and stomach [10], pancreatic cells [11], lymphocytes [12], neural progenitor cells (NPCs) [13], [14], [15], human keratinocytes [16], [17] and human CD34^+^ cells [18], have been reprogrammed. Among the cell types tested, NPCs are particularly interesting. They can be reprogrammed by ectopic expression of two factors (Oct4 and Klf4) or even one factor (Oct4) alone [14], [15], providing a unique tool to analyze reprogramming events at a molecular level. Using NPCs, Silva *et al.* delineated two phases in the reprogramming process (pre-pluripotency and ground state pluripotency) and demonstrated that dual inhibition (2i) of mitogen-activated protein kinase (MAKP) signaling and glycogen synthase kinase-3 (GSK3) signaling combined with the self-renewal cytokine leukemia inhibitory factor (LIF) promotes pre-pluripotent cells to ground state pluripotency [19]. While new techniques and insights are discovered at a rapid speed, the process by which differentiated adult cells are converted into a fully pluripotent state following forced expression of reprogramming factors remains obscure.

Generally, reprogramming occurs over many days in most cell types, and it seems that fewer factors are introduced, longer time is required for reprogramming to occur [15]. Furthermore, it takes significantly longer time to reprogram human cells than mouse cells. To delineate the molecular mechanism, a reproducible culture system for iPS cell derivation is critical. With current methodologies, mouse embryonic fibroblast (MEF) cells have been used as a feeder layer to derive both mouse and human iPS cells. However, there are certain drawbacks in MEF cells for derivation of iPS cells, in particular of human iPS cells. For instance, MEF cells are usually made of fibroblasts from the mouse embryos at embryonic day 13.5 and only cells at early passages (p2 to p3) are used as feeders for derivation and culture of embryonic stem (ES) and iPS cells. Their limited proliferation capacity makes it necessary to prepare MEF cells often, creating batch-to-batch variability and reducing reproducibility in derivation of iPS cells. Furthermore, the proliferation-inactivated MEF cells usually function well only for 5–7 days under ES or iPS cell culture conditions. However, approximately 10 days are needed for mouse cells and a longer time is required for human cells to be reprogrammed. The short life span of MEF cells has necessitated repeated passaging for reprogramming cells onto new feeders, making mechanistic dissection of the reprogramming process difficult. Thus, it is desirable to have feeder cells which can survive for an extended period of time to support the derivation of iPS cells after irradiation. Previously, we successfully established one mouse ES cell line on human foreskin-derived fibroblast (HFF) cells, which could be passaged at least 30 times, retaining an ability to support ES cell pluripotency (Li, *et al*. unpublished data). In light of the observation that iPS cell derivation takes place under the same culture conditions used for ES cells [20], we hypothesized that these human feeder cells could offer a stable tool for defining molecular hallmarks during conversion of differentiated somatic cells to the pluripotent state.

In this study, we established mouse iPS cell lines from NPCs of neonatal EGFP-transgenic mice on the HFF feeder cells via viral transduction of 4 factors, combined with application of 2i. Uniquely, the derivation and maintenance of the iPS cells were achieved in the absence of exogenous LIF. Mouse NPCs were efficiently reprogrammed into typical iPS cells, where endogenous expression of pluripotency markers was fully activated and transgenes were completely silenced. The cells displayed the typical mouse ES cell morphology and self-renewed *in vitro* for a long period of time (more than 30 passages). In suspension culture, they formed embryoid bodies (EBs), which contained various cell types originating from three embryonic germ layers. Teratomas were produced after the iPS cells were transplanted to immune-deficient mice. Importantly, we obtained live chimeric mice from these iPS cells with high degree of chimerism. The iPS cells contributed to multiple organs and tissues, including the germ cells in the testis. Thus, the study, for the first time, reports mouse iPS cell lines generated on human feeder cells, providing a reliable tool for further dissecting the molecular mechanisms of nuclear reprogramming.

## Results

### Primary culture of mouse NPCs

**Figure 1:**
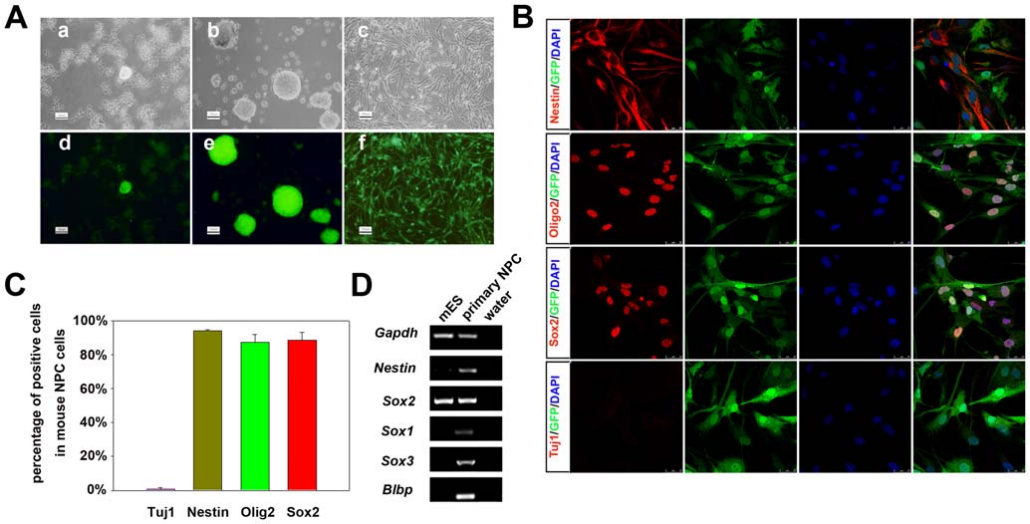
Generation and characterization of NPCs from neonatal mice. (A) Derivation of mouse NPCs. (a) Primary culture of NPCs of neonatal mice carrying the EGFP transgene. (b) Neural spheres were formed in suspension culture. (c) NPCs grew out of neural spheres in adhesive culture. (d)–(f) Corresponding EGFP images of (a)–(c). Scale bars are 50 µm in (a) and (d), 100 µm in (b), (c), (e) and (f). (B) Immunofluorescence staining with antibodies against Sox2, Nestin, Olig2 and Tuj1. Green color indicates EGFP-transgene. Scale bars are 25 µm. (C) Quantitative data of Sox2, Nestin, Olig2 and Tuj1 positive cells in primary NPCs were shown from three independent experiments. (D) RT-PCR results of early NPC markers were shown. Mouse ES (mES) cells were used as a negative control.

Based upon reports that mouse NPCs could be converted to the pluripotent state with higher efficiency than fully differentiated somatic cells [19] or with fewer reprogramming factors [13], [14], [15], we utilized NPCs as recipient cells to test the feasibility of using HFF cells in derivation of iPS. To generate primary NPCs, we minced the brain tissues from neonatal mice carrying the EGFP-transgene and digested them into single cells, which were plated onto low-attachment dishes for neurosphere formation (Figure 1A). Primary neurospheres were then collected and replated onto matrigel-coated tissue culture dishes for adherent culture in the same medium for another 2–3 days. Typical NPCs grew and migrated out of the spheres. The NPCs in the monolayer culture could be passaged 1–2 times before iPS cell induction. To characterize the NPCs, immunostaining experiments were conducted with antibodies against NPC markers, including Sox2, Nestin and Olig2. As illustrated in Figures 1B and 1C, a majority of the NPCs expressed Sox2, Nestin and Olig2, while much fewer cells expressed neuronal marker TuJ1. Moreover, RT-PCR analysis indicated expression of *Nestin, Sox1, Sox2, Sox3* and *Blpb* in NPCs (Figure 1D). This population of NPCs was used as starting cells for iPS induction in the following experiments.

### Derivation of mouse iPS cells on HFF cells

**Figure 2:**
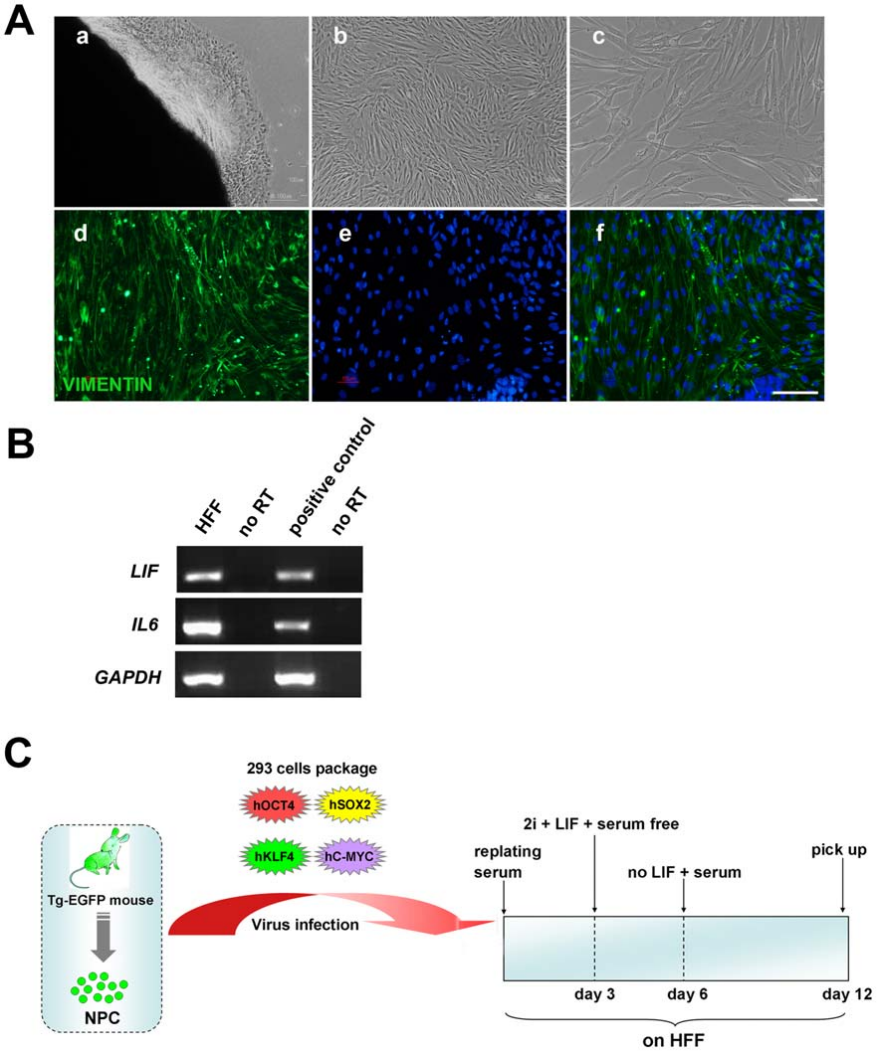
Derivation of HFF cells and the flow diagram for iPS induction. (A) Derivation of HFF cells. (a) HFF cells displayed homogeneous morphology when they grew and migrated out from foreskin tissue clumps. (b) HFF cells at early and (c) late passages. (d–f) Immunofluorescence staining of HFF cells with the antibody against VIMENTIN. Scale bars are 100 µm. (B) RT-PCR results of HFF cells using human *LIF* and *IL*6 specific primers. (C) Flow diagram of iPS induction in this study. Tg, transgene.

To derive HFF cells, small tissue clumps of the foreskin were plated onto culture dishes. HFF cells grew and migrated from the periphery of the tissue clumps (Figure 2A). From early passages (P2) to late passages (P31), they displayed homogeneous fibroblastic morphology and expressed VIMENTIN, which is considered as a fibroblast marker. After irradiation, they were able to maintain the typical morphology for up to two weeks, a capacity which is especially advantageous for iPS cell derivation. We also collected the RNA samples from HFF cells and examined expression of LIF and IL6. RT-PCR analysis revealed that the HFF cells could produce both LIF and IL6 (Figure 2B).

To derive iPS cells from primary NPCs, EGFP-positive NPCs were infected with the retrovirus containing 4 factors. As illustrated in Figure 2C, on the second day, the infected NPCs were replated onto irradiated HFF cells in the HFF medium. Two days later, 2i and N2B27 plus LIF replaced the HFF medium and the culture lasted for another three days. Subsequently, the culture medium was returned to the HFF medium without LIF. Following this scheme, ES cell-like colonies appeared gradually and were picked up on day twelve. Twelve colonies were picked up, of which independently picked single clones 11.1 and 4.1 were propagated continuously on HFF and fully characterized.

### The iPS cells are fully reprogrammed

**Figure 3:**
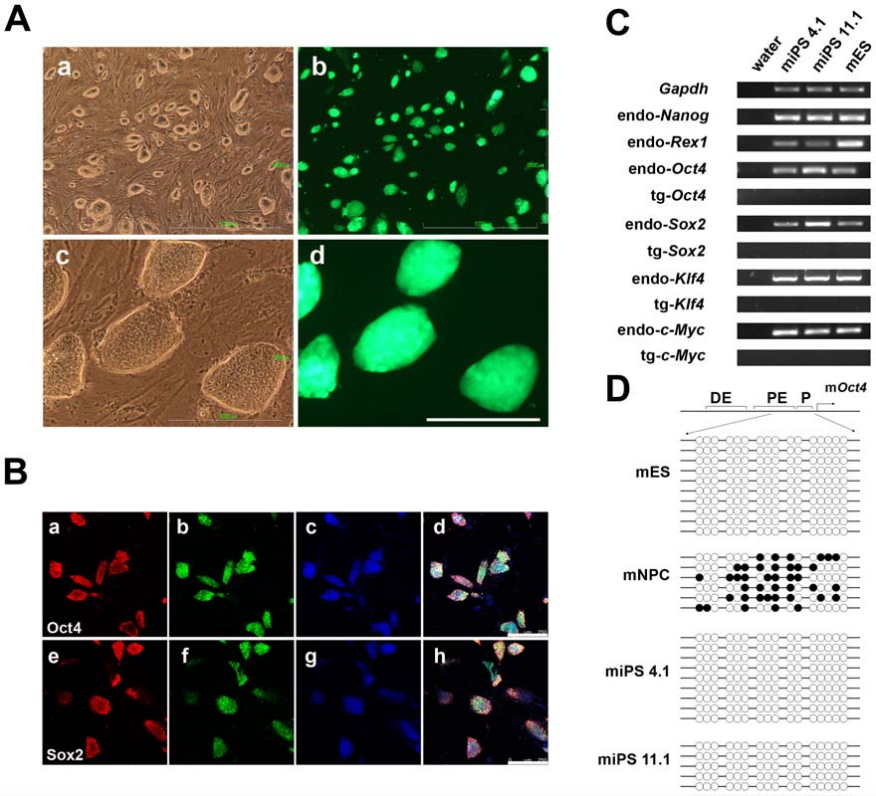
Characterization of iPS cells from NPCs. (A) Typical morphology of the iPS cells of line 11.1 stably propagating *in vitro* for 30 passages. (a) Low magnification of iPS cells of line 11.1, (b) EGFP image of (a), (c) high magnification of iPS cells of line 11.1, (d) EGFP image of (c). Scale bars are 1000 µm in (a) and (b), 200 µm in (c) and (d). (B) Immunofluorescence staining of iPS cells of line11.1 cells at passage 5 with Oct4 (a)–(d) and Sox2 (e)–(h) antibodies. Scale bars are 250 µm. (C) RT-PCR results using specific primers against transgenic 4 factors and endogenous pluripotency markers. Endo, endogenous genes; tg, transgenes. (D) Methylation status of the endogenous *Oct4* promoter was analyzed by bisulfite sequencing. Solid circles denote methylated sites and hollow circles denote demethylated sites.

The iPS cells displayed typical ES cell-like morphology, including a high nucleus-to-cytoplasm ratio, prominent nucleoli and clear boundaries among cells. All of these iPS cells expressed high levels of EGFP in continuous culture (Figure 3A), validating their origin of EGFP-transgenic mice. They proliferated for a prolonged period of time (30 passages) *in vitro* maintaining a normal 40 XY chromosome number (supporting information Figure S1) and expressed Oct4 and Sox2, as revealed by immunofluorescence staining (Figure 3B). Genomic PCR analysis revealed integration of 4 transgenic factors in iPS cells (supporting information Figure S2). Furthermore, data from RT-PCR analysis showed that transgenic 4 factors were completely silenced and endogenous pluripotency markers Oct4, Sox2, Rex1 as well as Nanog were successfully activated (Figure 3C). Like mouse ES cells, iPS cells from both line 11.1 and line 4.1 displayed complete demethylation at the endogenous *Oct4* promoter, indicating that full reprogramming occurred. On the other hand, parental NPCs showed approximately 50% methylation at the *Oct4* promoter (Figure 3D).

### Differentiation of iPS cells *in vitro*

**Figure 4:**
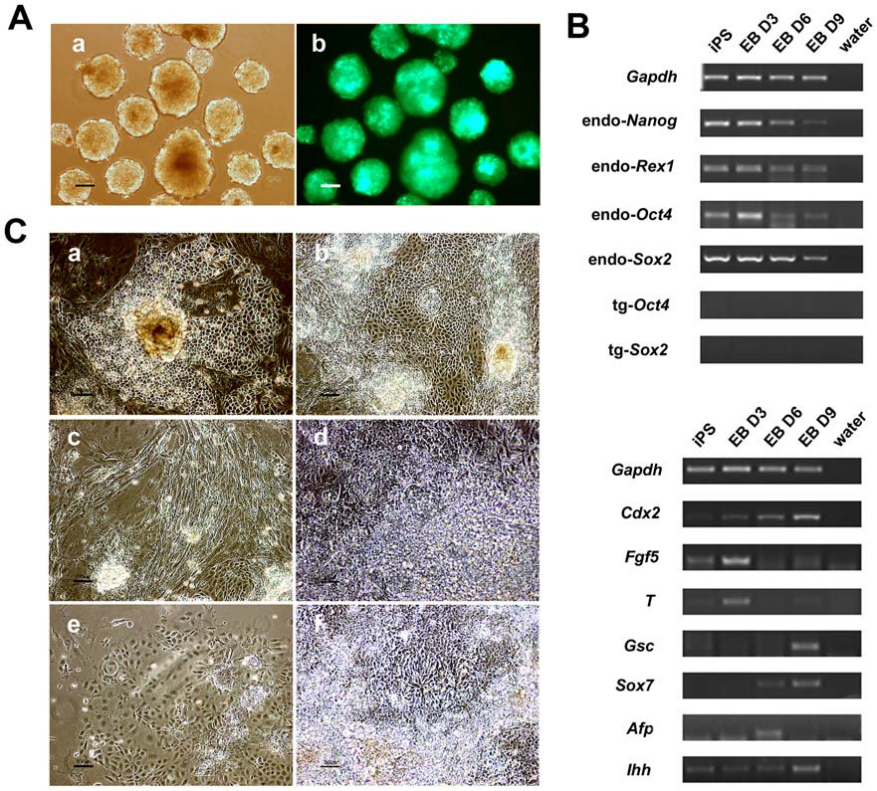
Spontaneous differentiation ability of iPS cells. (A) Well-shaped EBs could be formed in suspension culture (a) and maintain expression of EGFP (b). Scale bars are 100 µm. (B) During EB formation processes, various pluripotency markers and differentiation markers were determined using RT-PCR. (C) When iPS cells were cultured in the absence of the HFF cells, various differentiated cells could be observed, such as definitive endoderm-like cells (a) and (b), fibroblast-like cells (c), cardiomyocytes (d), epidermis-like cells (e) and neural rosettes (f). Scale bars are 100 µm in (a), (b), (c) and (e), 50 µm in (d) and (f).

To examine the differentiation potential, we suspended the iPS cells in low-attachment culture dishes for EB formation. The iPS cells from both line 11.1 and line 4.1 formed well-shaped EGFP-positive EBs (Figure 4A). RT-PCR analysis of RNA samples from EBs on days 3, 6 and 9 revealed a gradual decrease in expression of endogenous pluripotency markers such as *Nanog*, *Rex1*, *Oct4* and *Sox2* as compared to their expression in undifferentiated iPS cells (Figure 4B, upper panel). Notably, no reactivation of transgenic *Oct4* and *Sox2* was detected. Simultaneously, expression of markers for major developmental lineages, including trophectoderm (*Cdx2*), ectoderm (*Fgf5*), mesoderm (*T*) and endoderm (*Gsc, Sox7* and *Afp*), was activated (Figure 4B, bottom panel). Moreover, we cultured iPS cells on gelatin-coated dishes without HFF cells. Under such conditions, various differentiated cells were found, including pulsatile cardiomyocyte cells, fibroblast-like cells (mesoderm), definitive endoderm-like cells (endoderm), neural rosettes and epidermis-like cells (ectoderm) (Figure 4C).

**Figure 5:**
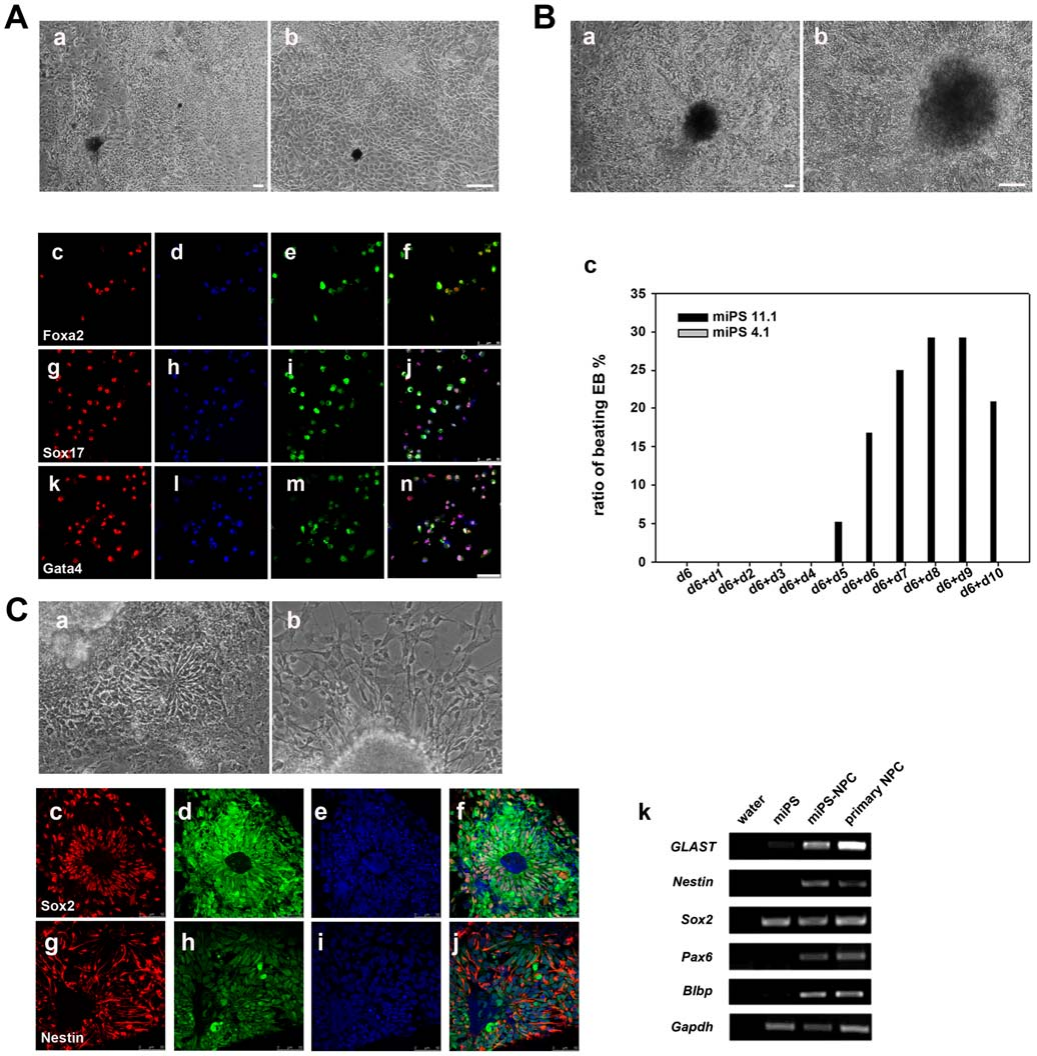
Directed differentiation of iPS cells towards definitive endoderm, cardiomyocyte and neural lineages. (A) A low concentration of FBS in combination with a high concentration of recombinant Activin A could induce iPS cells of line 11.1 into a definitive endoderm lineage. (a) Low magnification and (b) high magnification of differentiated cells are shown. Immunofluorescence staining of definitive endoderm markers (Foxa2, Sox17 and Gata4) was carried out (c)–(n). Expression of EGFP indicates the EGFP transgenic origin. Scale bars are 100 µm in (a) and (b), 50 µm in (c)–(n). (B) Beating foci of cardiomyocytes were observed. (a) Low magnification and (b) high magnification of beating foci were shown. (c) The ratio of beating EBs in directed differentiation of iPS cells of line 11.1 into cardiomyocytes was shown. The representative results of two independent experiments are shown. Scale bars are 100 µm. (C) Directed differentiation of iPS cells of line 11.1 into neural lineages. (a) Typical neural rosettes, (b) neural cells migrating from adhesively cultured neurospheres. (c)–(f) Immunofluorescence staining using Sox2 antibody. (g)–(j) Immunofluorescence staining using Nestin anbibody. Expression of EGFP indicates the EGFP transgenic origin. (k) RT-PCR results of early NPC markers for iPS cell line 11.1 derived NPCs. Scale bars are 100 µm.

In addition to spontaneous differentiation, we evaluated the ability of iPS cells to differentiate into three embryonic germ layers using directed differentiation protocols. For definitive endoderm differentiation, spontaneously formed EBs on day 6 were plated onto matrigel-coated dishes and maintained in a low concentration of fetal bovine serum (FBS) in combination with a high concentration of recombinant Activin A. Scale-like cells appeared (Figure 5A), which were positive for definitive endoderm markers such as Foxa2, Sox17 and Gata4. To induce cardiomyocyte differentiation, compact and well-shaped EBs formed in suspension culture containing 10% of FBS for 6 days were plated in 24-well plates in the presence of 15% of FBS. Contractile foci were found and counted each day (Figure 5B and supporting information Movie S1). The ratio of beating EBs for iPS cell line 11.1 increased with the length of differentiation and reached approximately 30% after plating for 8 days, falling down after 10 days. Interestingly, we did not find any beating EBs in iPS line 4.1 under the same differentiation conditions (Figure 5B). Nevertheless, no obvious differences in expression of the early cardiac markers and transcription factors (*Gata4*, *Mef2c*, *Hind 1, Nkx2-5* and *beta-Mhc*) were found between two iPS cell lines (supporting information Figure S3). Furthermore, neural induction was achieved by addition of retinal acid (RA, 2 µm) to the EB culture of iPS cells at day 3. After 3 days of RA treatment, the EBs were plated on the matrigel-coated culture dishes in the N2B27 medium for several days. Typical neural rosettes and neuronal cells could be detected for both lines of iPS cells. Immunostainig with Sox2 and Nestin antibodies and RT-PCR for early NPC markers further demonstrated the neural lineage differentiation (Figure 5C). Taken together, the evidence shows that NPC-derived iPS cells can differentiate spontaneously or be induced into specific lineages reproducibly *in vitro*.

### Differentiation of iPS cells *in vivo*

**Figure 6:**
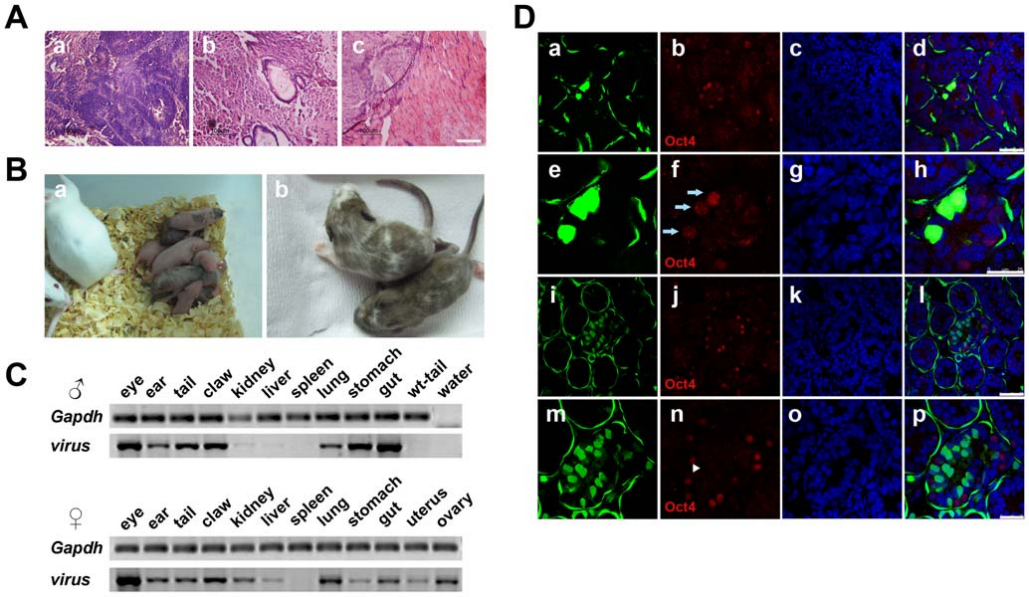
Differentiation of iPS cells *in vivo*. (A) Teratomas were histochemically analyzed (HE staining). Typical neural rosettes (a), intestines (b) and muscles (c) were found. Scale bars are 100 µm. (B) Chimeric mice from iPS cells of line 11.1. (a) Neonatal chimeric mice, (b) two-week old chimeric mice. Black color indicates contribution of iPS cells of line 11.1. (C) Genomic DNA from various organs of two-week old male and female chimeric mice was analyzed using genomic PCR and virus-specific primers. (D) Testes of the male chimeric mouse were sliced transversely and analyzed for EGFP and Oct4 expression. The iPS cells expressing EGFP developed to spermatogonial stem cells. (a)–(d) and (i)–(l), low magnification; (e)–(h) and (m)–(p), high magnification for immunofluorescence staining of Oct4. Arrows indicate Oct4 positive and EGFP expressed spermatogonial stem cells. Arrow heads indicate Oct4 negative and EGFP expressed spermatocytes. Scale bars are 50 µm in (a)–(d) and (i)–(l), 25 µm in (e)–(h) and (m)–(p).

To test the differentiation potential of iPS cells *in vivo*, we injected the undifferentiated iPS cells into SCID-beige mice for teratoma formation. Four weeks after injection, well-shaped teratomas were found. Histochemical analysis indicated the existence of tissues from all three germ layers, such as neural epithelial cells (ectoderm), intestines (endoderm) and muscle (mesoderm) (Figure 6A). Our results show that iPS cells of both line 11.1 and 4.1 at either early (P5) or late (P15) passages could produce teratomas containing derivatives of three embryonic germ layers. There was not any discernible difference between these two iPS cell lines in the ability to form teratomas. Since chimera and germline contribution assays are considered as gold standards for the identity of genuine ES or iPS cells, we injected iPS cells of both line 11.1 and line 4.1 into blastocysts and obtained 12 live chimeric pups from cells of line 11.1 and 2 from line 4.1, respectively. The pups had marked color chimerism ranging from 10% to 90% (Figure 6B, Table 1 and supporting information Figure S4). To further verify their incorporation into host organs, we sacrificed two-week old chimeric mice. PCR analysis of the genomic DNA demonstrated extensive contribution of injected iPS cells into various organs including the eyes, ears, tail, claws, kidneys, liver, lungs, stomach and guts. For females, the exogenous factor integration was also confirmed in the uterus and ovary. Interestingly, we did not find any viral integration in the spleen of either male or female chimera tested (Figure 6C). In addition, the contribution of iPS cells to the livers of chimeric mice was evaluated by immunofluorescence staining using antibodies against CD31 (a marker of vascular endothelial cells) and AFP (a marker of liver cells). Coexpression of CD31 and EGFP or AFP and EGFP was found (supporting information Figure S5), indicating that injected iPS cells differentiated and incorporated into endothelial and hepatic cells in the liver. Finally, testes of the male chimeric mouse were transversely sliced and stained with the antibody against Oct4 to examine the contribution of iPS cells to the germline. Confocal images of sections of the seminiferous tubules illustrated the existence of various iPS cell-derived EGFP-positive cells, including flattened fibroblast cell-like myoid at the outermost layer of the tubules, spermatogonial stem cells with spherical nuclei closest to the basement membrane and spermatocytes with distinctive chromatin closer to the lumen than spermatogonial stem cells (Figure 6D). Among these green cells, there were Oct4-positive spermatogonial stem cells and Oct4-negative spermatocytes, providing evidence of the contribution of injected iPS cells to the germline.

**Table 1:** Efficiency of making chimera mice from iPS cell lines.

iPS lines	Blastocysts Injected	Chimeric/Total	Chimaerism (%)	Germline
iPS 11.1	58	12/23	20–90	Yes
iPS 4.1	16	2/9	<10	ND

ND, not determined.

## Discussion

To our knowledge, this is the first report of generation and maintenance of mouse iPS cells on human feeder cells without exogenous LIF. There are several advantages of using HFF cells over MEF cells. The first is the ability to reproducibly support deriving iPS cells, which is important for mechanistically dissecting the reprogramming process. In contrast to MEF cells, HFF cells can proliferate robustly for at least 30 passages *in vitro* and maintain the ability to support derivation and culture of iPS cells. They can be easily frozen and thawed, ensuring production of a large number of qualified feeder cells and allowing comparison of the variables influencing reprogramming efficiency under relatively stable conditions. Secondly, considering that direct reprogramming is a slow and inefficient process, especially for human cells, it is desirable to have feeder cells which can last long enough for reprogramming to proceed. Our HFF cells can remain healthy for 2 weeks after irradiation to stop their proliferation. Compared to MEF cells, which can be maintained for only 5–7 days after irradiation, HFF cells as a feeder layer avoid frequent passage and allow the maintenance of established cell-cell interaction during the reprogramming process. Also, it is beneficial for continuous observation of the reprogramming process, in particular at single colony or even single cell levels. Thirdly, HFF cells are more tolerant to certain reagents added to the culture medium. Indeed, we attempted to reprogram the NPCs initially on MEF cells. Seventy-two hours after retroviral delivery of 4 factors into the NPCs, ES cell-like colonies could be detected. However, as reported by Silva *et al*. [19], most of these colonies were pre-pluripotent cells. To promote the partially reprogrammed NPCs to fully pluripotent status, we adapted the 2i strategy [19]. Unfortunately, inclusion of the 2i in our culture system resulted in massive death of irradiated MEF cells plated at a relatively low density, suggesting that the 2i strategy might not be compatible with irradiated MEF cells. In contrast, addition of the 2i into HFF cell culture did not affect their survival. Thus, various reagents and compounds can be tested for their potential roles in reprogramming events. Lastly, it is easy to transfect the HFF cells and select stably transfected cells. We have established HFF cell lines which stably express resistant genes against neomycin, puromycin and hygromycin (data not shown). These cell lines offer convenient tools for experiments where transgenes and selection are required.

In this study, we established two independent iPS cell lines, line 11.1 and line 4.1. Cells from both lines satisfied standard criteria of mouse iPS cells. However, they exhibited differential tendencies when they were cultured under differentiation conditions. For instance, cells from line 11.1 could be efficiently induced to form contractile cardiomyocytes, while iPS cells from line 4.1 failed to do so under the same conditions. On the other hand, cells of line 4.1 differentiated into endoderm lineages more easily than cells of line 11.1 (data not shown). Therefore, it is necessary to establish multiple independent iPS cell lines from individual donors to ensure efficient generation of various types of functional cells for future application in the treatment of diseases. As for the reasons of the differential differentiation tendency observed in these two iPS cell lines, it could result from differences in their reprogramming dynamics or in intergration sites of the transgenes. Interestingly, cells from both iPS cell lines expressed early cardiac differentiation markers and transcription factors, implicating that there could be a lack in functional cardiomyocyte maturation in line 4.1. Nevertheless, cell lines with varying differentiation potential might also aid in study of molecular regulation of differentiation processes.

The third interesting advance made in this study is the discovery that mouse iPS cell lines could be established and maintained for 30 passages on HFF cells without exogenous LIF. Our unpublished data show that HFF cells can support derivation and culture of mouse ES cells in an undifferentiated state without exogenous LIF for more than 30 passages. In contrast, the same mouse ES cells cultured on MEF cells began to differentiate after 2 passages in the absence of LIF (data not shown). Although HFF cells could secrete LIF and IL6 into the culture medium, we could not exclude the possibility that the HFF cells might provide unique factors or niches for ES and iPS cells to maintain self-renewal. Further investigation is required to understand the genetic regulation underlying the distinct properties. It is also worthy to mention that human ES cells and iPS cells could be maintained on the HFF cells with the conventional human ES culture medium (Li *et al*, unpublished data). Therefore, HFF cells might serve as feeder cells to support derivation of xeno-free human iPS cells.

In summary, two independent genuine mouse iPS cell lines were generated from neonatal NPCs on HFF cells in the absence of exogenous LIF. The study demonstrates that HFF cells are very useful tools for iPS cell derivation and culture condition optimization. In addition, their independence of LIF for iPS cell culture provides an opportunity to further understand the ground state of ES cell self-renewal proposed by Ying *et al*. [21]. The cell lines established in this study are freely available for the scientific community.

## Materials and Methods

### Derivation and culture of HFF cells

Human discarded foreskin tissues were obtained from the Shanghai Children's Center upon the approval from the Ethical Review Board of the Institute of Health Sciences and after obtaining the written informed consent from parents of the child participants. Clinically circumcised foreskin tissues were washed thoroughly in PBS containing 500 U/ml penicillin (Invitrogen) and 500 µg/ml streptomycin (Invitrogen) and cut into small pieces of approximately 0.5×0.5 cm^2^. The tissue clumps were then seeded into 100 mm culture dishes and cultured at 37°C with 5% CO_2_. After fibroblast cells grew out and reached 90% confluence, they were passaged using TrypLE Express (Invitrogen). The culture medium for HFF cells contained DMEM (Invitrogen), 10% human serum (Sigma), 1% nonessential amino acids (NEAA), 2 mM L-glutamine (Invitrogen), 100 U/ml penicillin and 100 µg/ml streptomycin. For induction and culture of mouse iPS cells, HFF cells were passaged using trypsin and cultured in medium containing DMEM, 10% FBS (Hyclone), 1% NEAA (Invitrogen), 2 mM L-glutamine (Invitrogen), 100 U/ml penicillin and 100 µg/ml streptomycin. Confluent HFF cells were treated with irradiation of 55 Gy and plated onto culture dishes (1×10^5^ per 2.89 cm^2^).

### Primary culture of mouse NPCs

Mice were maintained in SPF spaces with a 14-h light and 10-h dark regime. All animal procedures were performed according to the guidelines approved by the Shanghai Jiao Tong University School of Medicine. Neonatal C57BL/6J mice carrying the EGFP transgene (Nanjing University, China) were sacrificed and sprayed with 75% ethyl alcohol. Under sterile conditions, the brain tissue of the mice was isolated and washed with PBS three times, then cut into small pieces. Brain tissue clumps were dissociated with 0.05% trypsin/EDTA (Gibco) at 37°C for 5 minutes, and filtered with a 70 µm cell strainer (BD). The cells were collected and centrifuged at 1000 g for 3 minutes. The precipitated cells were resuspended in the serum-free medium containing DMEM/F12 (Invitrogen), B27 (2%) (Invitrogen), bFGF (20 ng/mL) (Invitrogen) and EGF (20 ng/mL) (R&D), and plated onto a 60 mm low-attachment dish. Then cells were cultured at 37°C with 5% CO_2_ for neurosphere formation. Primary neurospheres could be observed from the second day, and the medium was replaced every day. After 6–7 days of suspension culture, the neurospheres were replated onto matrigel-coated 60 mm tissue culture dishes for adherent culture in the same medium for another 2–3 days. The adherent neurospheres were dissociated with 0.05% trypsin/EDTA into single cells and passaged for monolayer culture. The NPCs in mono-layer culture for 2–3 passages were characterized by immunostaining and could be frozen until use.

### Retrovirus production and transduction

For retrovirus production, human cDNAs for *OCT4*, *SOX2*, *KLF4* and *C-MYC* in the pMXs vector from Addgene (5 µg each), p-Gag-Pol (3.5 µg) and p-VSV-G (1 µg) were co-transfected into 293 cells with the Fugene 6 kit (Roche). Virus-containing supernatants were collected forty-eight hours after transfection, passed through a 0.45 µm filter (Millipore) and concentrated by ultracentrifugation at 100,000 g for two hours at 4°C. The viral pellets were resuspended in DMEM (Hyclone) without additives and then aliquoted into cryoviral tubes and stored at −80°C. For viral transduction, 2×10^5^ NPCs were seeded on matrigel-coated 60 mm dishes. Six hours later, a mixture of four viruses was added to the medium for infection overnight with 8 µg/ml polybrene (Sigma).

### Derivation and culture of mouse iPS Cells

Twenty-four hours after infection, NPCs were replated onto irradiated HFF cells in HFF cell culture medium. Two days later, the medium was changed to the N2B27 medium supplemented with the 2i plus LIF [19]. Three days later, the medium was replaced with the DMEM medium containing 10% FBS, 100 U/ml penicillin, 100 µg/ml streptomycin, 0.1 mM β-mercaptoethanol (Sigma) and 2 mM L-glutamine. On day twelve, single colonies were picked up and propagated on HFF cells without exogenous LIF. Mouse iPS cells were maintained with 90% the Glasgow Minimum Essential Medium (GMEM, Invitrogen) containing 10% FBS, 100 U/ml penicillin, 100 µg/ml streptomycin, 0.1 mM β-mercaptoethanol and 2 mM L-glutamine.

### RT-PCR

Total RNA was extracted from the cells using TRIzol (Invitrogen) and transcribed into cDNA using oligo (dT) 16 and ReverTra Ace reverse transcriptase (Toyobo). PCR reactions were carried out with 1 µl of cDNA template, 250 nM of each primer, 200 µM of dNTP mixture, and 1 U of Taq DNA polymerase in a volume of 20 µl. Samples were amplified in a thermocycler. The primer information is provided in supporting information, Table S1.

### Immunofluorescence staining

Cells and tissue sections were stained as previously described [22]. Information for antibodies used in this study is provided in supporting information, Table S2. All images were captured using a confocol microscope (Leica).

### Chromosome number counting

Chromosome number counting was carried out with iPS cells at passage 18. The cells were incubated with 0.2 µg/ml demecolcine (Sigma) for 3 hours and then resuspended in 0.075 M KCl for 30 minutes at 37°C. Hypotonic solution-treated cells were fixed in methanol: acetic acid (3:1 in volume) for 30 minutes at room temperature, dropped onto pre-cleaned slides and stained with DAPI. More than ten metaphase spreads were counted.

### Bisulfite Sequencing

Genomic DNA from the iPS cells, ES cells and NPCs was restricted with EcoRV and treated with sodium bisulfite as previously described [23]. Treated DNA was subjected to nested PCR. Primer sequences are described in supporting information Table S1. The PCR products were cloned into the T-easy vector (Promega) and individual clones were sequenced.

### *In vitro* differentiation assay

For monolayer differentiation, confluent iPS cells were cultured in the HFF culture medium without any growth factors. Differentiated cells of all three germ layers were detected. For EB formation, iPS cells were digested with trypsin into single cells and cultured in suspension in low-attachment dishes. For cardiomyocyte differentiation, EBs were suspended for six days in DMEM plus 10% FBS. Well-shaped EBs were plated onto gelatin-coated dishes in DMEM plus 15% FBS. Beating foci were counted every day. For definitive endoderm differentiation, EBs suspended for six days were plated onto matrigel-covered dishes with a low concentration of FBS (0.5%) and a high concentration of recombinant Activin A (50 ng/ml) for five days. Differentiation into NPCs was induced as described previously [24].

### Teratoma formation

For teratoma formation, 5×10^6^ cells of early passage (P5) and late passage (P15) from each iPS cell line were harvested and injected intramuscularly into SCID-beige mice. Four weeks later, teratomas were harvested. Sections were processed with hematoxylin and eosin (HE) staining.

### Blastocyst injection and embryo transfer

Diploid blastocysts at 3.5 day post coitum (dpc) were collected from the uterus of superovulated ICR females mated with ICR males and kept in KSOM medium with amino acids until iPS cell injection. iPS cells were trypsinized, resuspended in DMEM without LIF, and kept on ice. A flat tip microinjection pipette with an internal diameter of 15–18 µm was used for iPS cell injection. Approximately ten to fifteen iPS cells were injected into the cavity of each blastocyst, all of which were kept in KSOM with amino acids until embryo transfer. Ten to fifteen injected blastocysts were transferred to each uterine horn of 2.5 dpc pseudo-pregnant ICR females.

### Analysis of chimeric animals

Male and female chimeric mice were sacrificed. Genomic DNA of various tissues and organs were extracted and analyzed by genomic PCR with virus primers as shown in supporting information Table S1. To examine germline transmission, the testes of a male chimeric mouse were transversely sliced. Contribution of iPS cells to the gonad was analyzed by EGFP expression and immunofluorescence staining.

## Supporting Information

Figure S1 Chromosome counting assay of mouse iPS cell. The iPS cells of line 11.1 had a normal 40 XY karyotype (ten metaphases were analysized).
(0.12 MB DOC)

Figure S2 Genomic PCR results of the transgenic four factors. Genomic DNA from cell line miPS 11.1 and mouse NPCs was used to carry out genomic PCR using specific primers of transgenic four factors. water was used as a control.
(0.17 MB DOC)

Figure S3 RT-PCR results of cardiomyocyte differentiation markers of iPS cells. The early cardiac markers and transcription factors (Gata4, Mef2c, Hind I, Nkx2-5 and beta-Mhc) were examined in iPS cell lines 11.1 and 4.1 during the cardiac differentiation process (differentiation day 0, day 3, day 6 and day 9). Gapdh was used as an internal control.
(0.22 MB DOC)

Figure S4 Chimeric mice from iPS cells of line 4.1. The chimaerism was estimated on the basis of the coat color. Donor cells of iPS cells of line 4.1 were from black mice (C57 BL/6J). The recipient blastocysts were obtained from white mice (ICR).
(3.40 MB DOC)

Figure S5 Integration of iPS cells in the liver of chimeric mice. The CD31 antibody-reactive cells (red, A and E) and AFP antibody-reactive cells (red, I and M) were detected specifically in the liver of chimeric mice from iPS cells of line 11.1. EGFP-positive cells (green, B, F, J and N) were the iPS cell-derived cells. Corresponding DAPI staining highlighting the nuclei is shown in panels C, G, K and O. The composite images are shown in panels D, H, L and P. The scale bars are 50 µm in A, B, C, D, I, J, K and L (low magnification), 25 µm in M, N, O (high magnification) and P, 10 µm in E, F, G and H (high magnification).
(6.04 MB DOC)

Table S1 Primers for RT- PCR and genomic- PCR.
(0.06 MB DOC)

Table S2 Antibodies used in immunofluorescence staining.
(0.03 MB DOC)

Movie S1 Beating Cardiomyocytes Differentiated from Mouse iPS Cell Line 11.1
(2.37 MB MOV)
