# Supplementary material for: Germline-Competent Mouse-Induced Pluripotent Stem Cell Lines Generated on Human Fibroblasts without Exogenous Leukemia Inhibitory Factor
Source: PLoS One. 2009 Aug 21;4(8):e6724. doi: 10.1371/journal.pone.0006724 (PMC2725300; doi:10.1371/journal.pone.0006724)
Supplement: Figure S1 — Chromosome counting assay of mouse iPS cell. The iPS cells of line 11.1 had a normal 40 XY karyotype (ten metaphases were analysized). (0.12 MB DOC) [file pone.0006724.s001.doc]

**
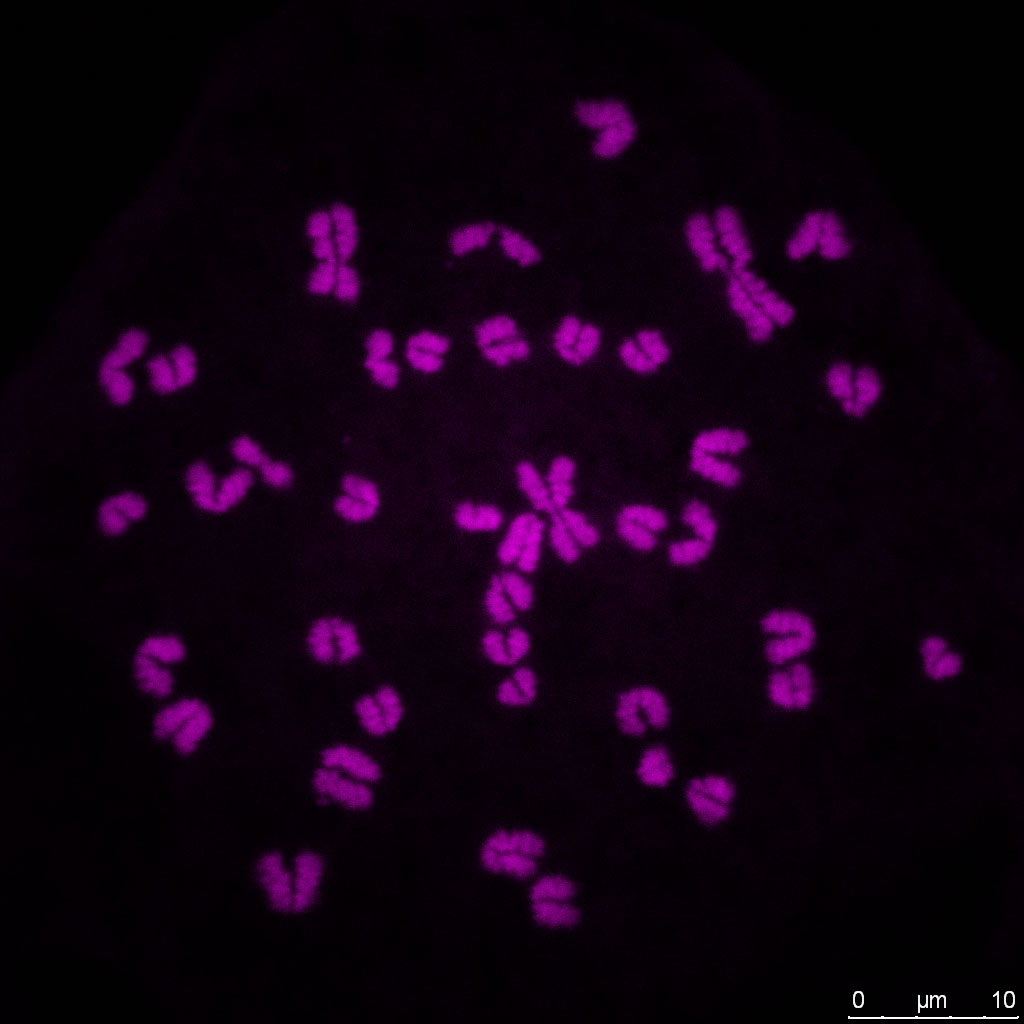
**

**Figure S1.** Chromosome counting assay of mouse iPS cell

The iPS cells of line 11.1 had a normal 40 XY karyotype (ten metaphases were analysized).
