# Supplementary material for: Germline-Competent Mouse-Induced Pluripotent Stem Cell Lines Generated on Human Fibroblasts without Exogenous Leukemia Inhibitory Factor
Source: PLoS One. 2009 Aug 21;4(8):e6724. doi: 10.1371/journal.pone.0006724 (PMC2725300; doi:10.1371/journal.pone.0006724)
Supplement: Figure S2 — Genomic PCR results of the transgenic four factors. Genomic DNA from cell line miPS 11.1 and mouse NPCs was used to carry out genomic PCR using specific primers of transgenic four factors. water was used as a control. (0.17 MB DOC) [file pone.0006724.s002.doc]

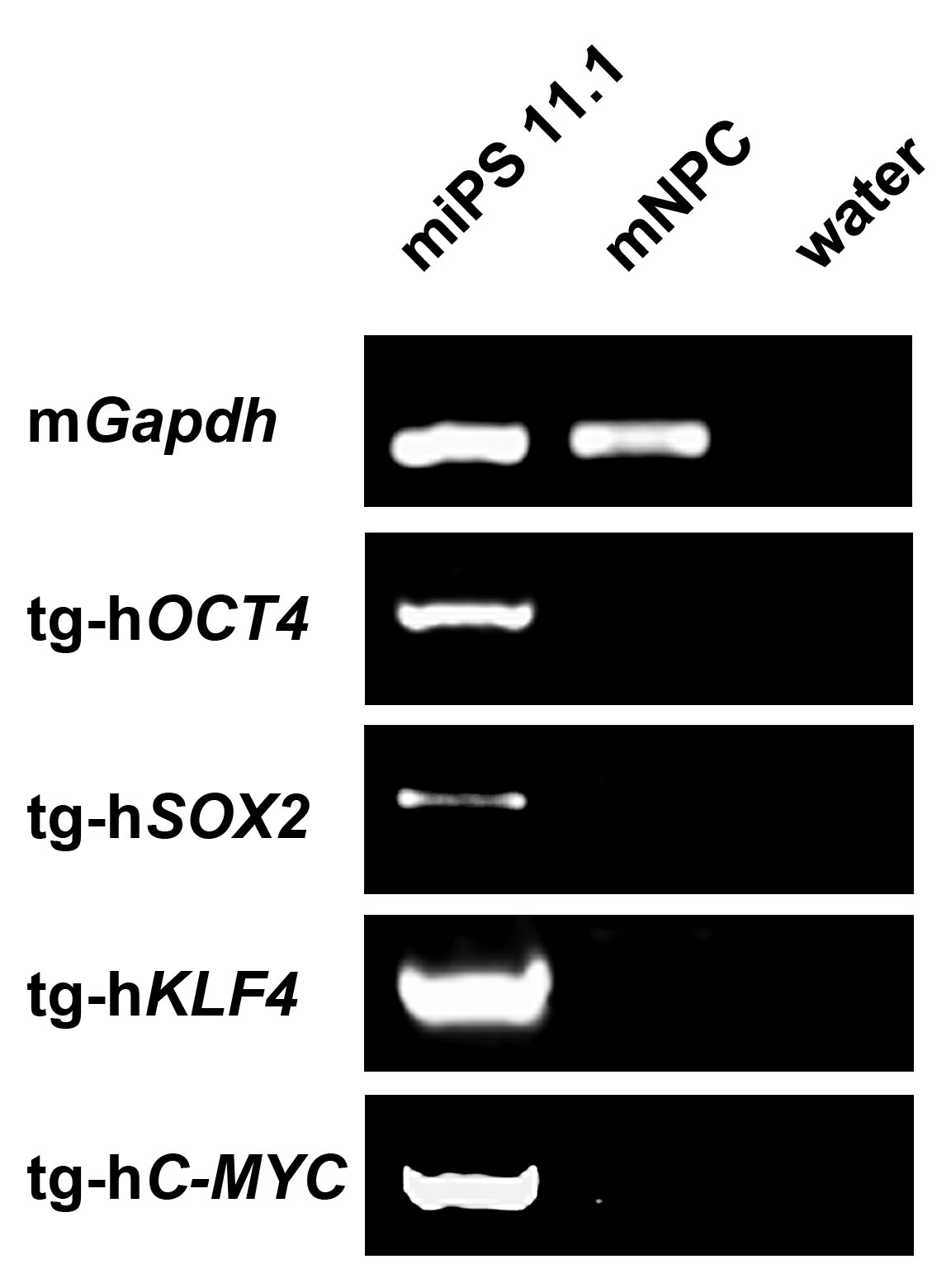


**Figure S2.** Genomic PCR results of the transgenic four factors

Genomic DNA from cell line miPS 11.1 and mouse NPCs was used to carry out genomic PCR using specific primers of transgenic four factors. water was used as a control.
