# Supplementary material for: Germline-Competent Mouse-Induced Pluripotent Stem Cell Lines Generated on Human Fibroblasts without Exogenous Leukemia Inhibitory Factor
Source: PLoS One. 2009 Aug 21;4(8):e6724. doi: 10.1371/journal.pone.0006724 (PMC2725300; doi:10.1371/journal.pone.0006724)
Supplement: Figure S3 — RT-PCR results of cardiomyocyte differentiation markers of iPS cells. The early cardiac markers and transcription factors (Gata4, Mef2c, Hind I, Nkx2-5 and beta-Mhc) were examined in iPS cell lines 11.1 and 4.1 during the cardiac differentiation process (differentiation day 0, day 3, day 6 and day 9). Gapdh was used as an internal control. (0.22 MB DOC) [file pone.0006724.s003.doc]

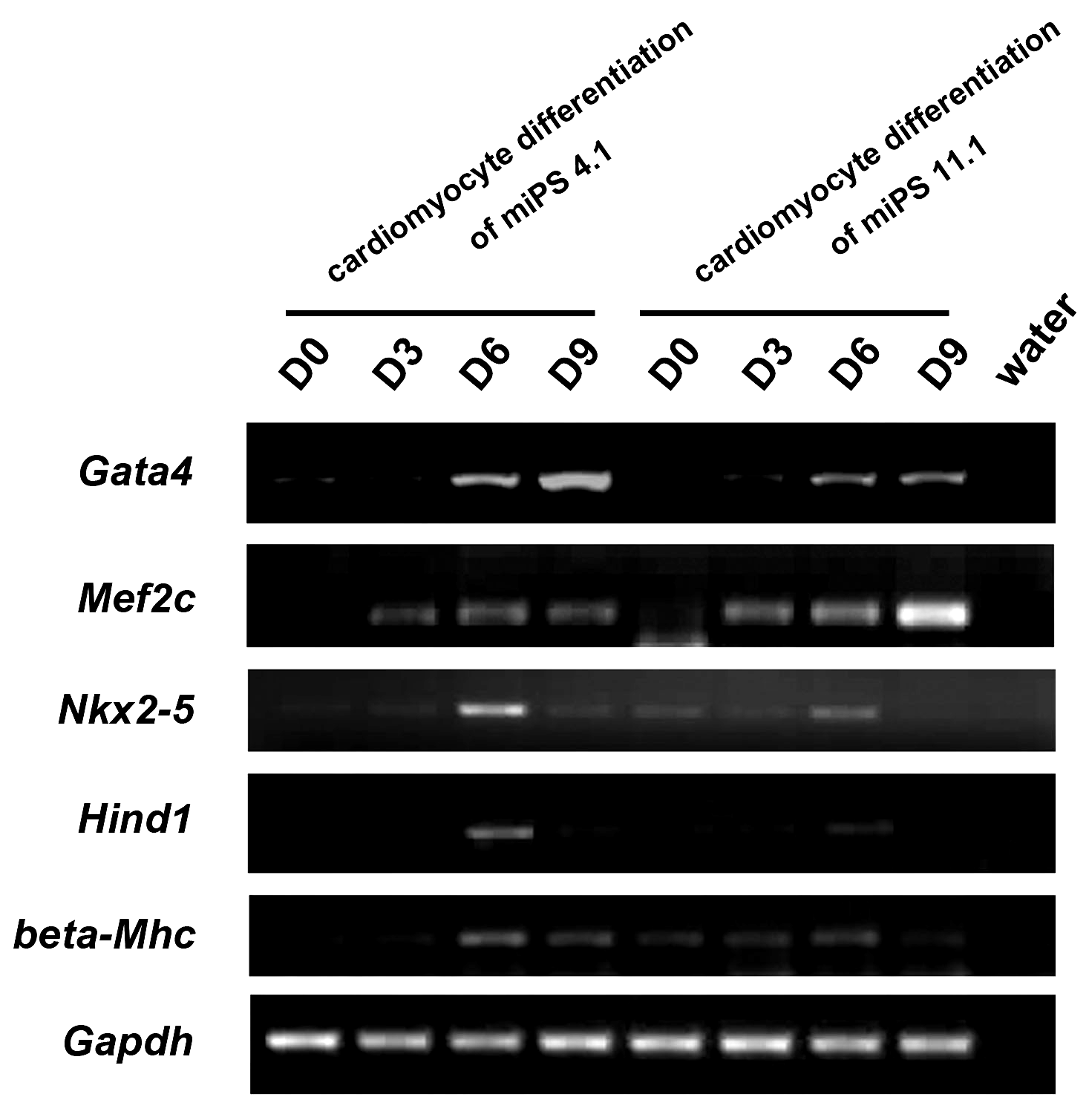


**Figure S3.** RT-PCR results of cardiomyocyte differentiation markers of iPS cells

The early cardiac markers and transcription factors (*Gata4*, *Mef2c*, *Hind I*, *Nkx2-5* and *beta-Mhc*) were examined in iPS cell lines 11.1 and 4.1 during the cardiac differentiation process (differentiation day 0, day 3, day 6 and day 9). *Gapdh* was used as an internal control.
