# Supplementary material for: Germline-Competent Mouse-Induced Pluripotent Stem Cell Lines Generated on Human Fibroblasts without Exogenous Leukemia Inhibitory Factor
Source: PLoS One. 2009 Aug 21;4(8):e6724. doi: 10.1371/journal.pone.0006724 (PMC2725300; doi:10.1371/journal.pone.0006724)
Supplement: Figure S4 — Chimeric mice from iPS cells of line 4.1. The chimaerism was estimated on the basis of the coat color. Donor cells of iPS cells of line 4.1 were from black mice (C57 BL/6J). The recipient blastocysts were obtained from white mice (ICR). (3.40 MB DOC) [file pone.0006724.s004.doc]

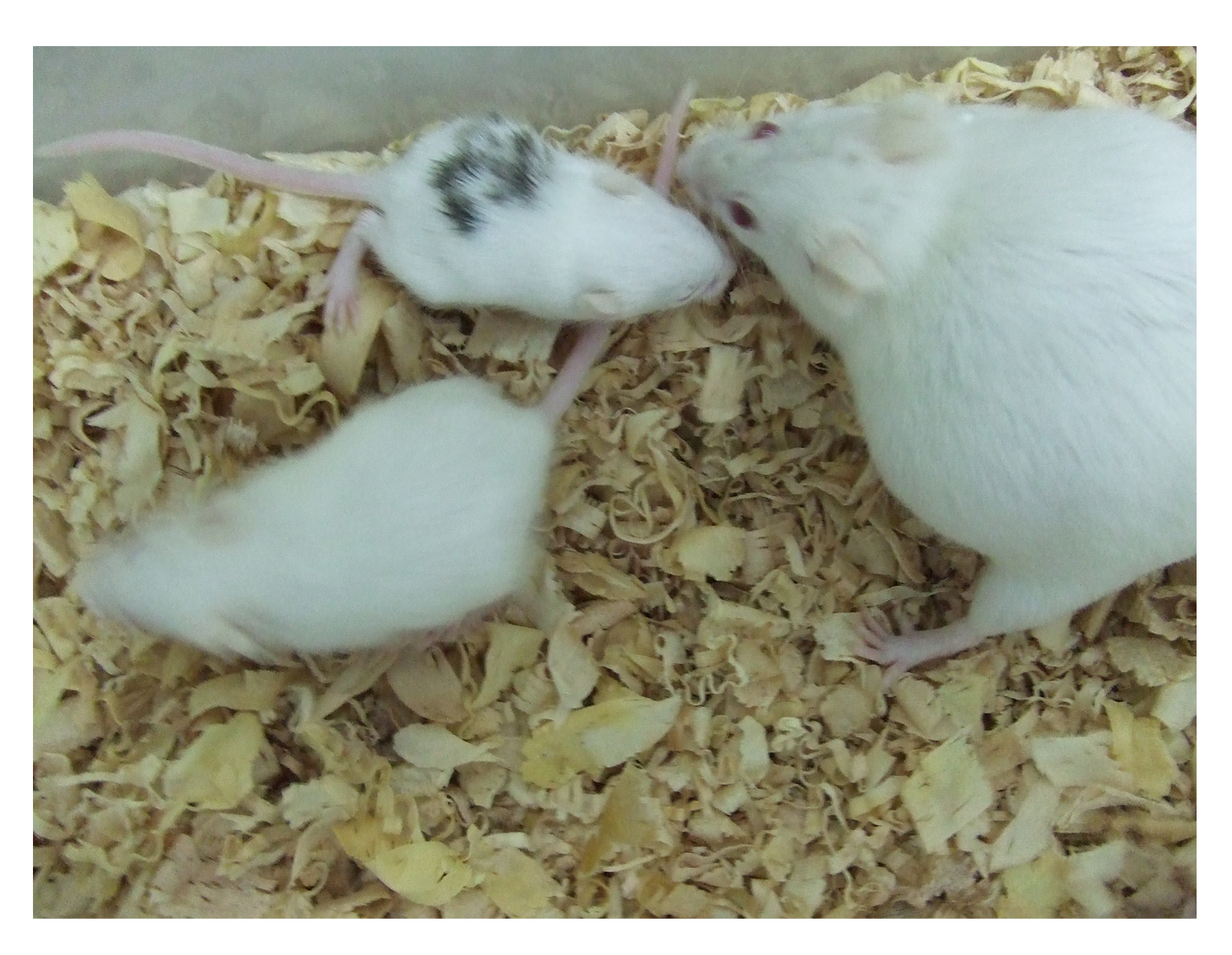


**Figure S4.** Chimeric mice from iPS cells of line 4.1

The chimaerism was estimated on the basis of the coat color. Donor cells of iPS cells of line 4.1 were from black mice (C57 BL/6J). The recipient blastocysts were obtained from white mice (ICR).
