# Supplementary material for: Germline-Competent Mouse-Induced Pluripotent Stem Cell Lines Generated on Human Fibroblasts without Exogenous Leukemia Inhibitory Factor
Source: PLoS One. 2009 Aug 21;4(8):e6724. doi: 10.1371/journal.pone.0006724 (PMC2725300; doi:10.1371/journal.pone.0006724)
Supplement: Figure S5 — Integration of iPS cells in the liver of chimeric mice. The CD31 antibody-reactive cells (red, A and E) and AFP antibody-reactive cells (red, I and M) were detected specifically in the liver of chimeric mice from iPS cells of line 11.1. EGFP-positive cells (green, B, F, J and N) were the iPS cell-derived cells. Corresponding DAPI staining highlighting the nuclei is shown in panels C, G, K and O. The composite images are shown in panels D, H, L and P. The scale bars are 50 µm in A, B, C, D, I, J, K and L (low magnification), 25 µm in M, N, O (high magnification) and P, 10 µm in E, F, G and H (high magnification). (6.04 MB DOC) [file pone.0006724.s005.doc]

**
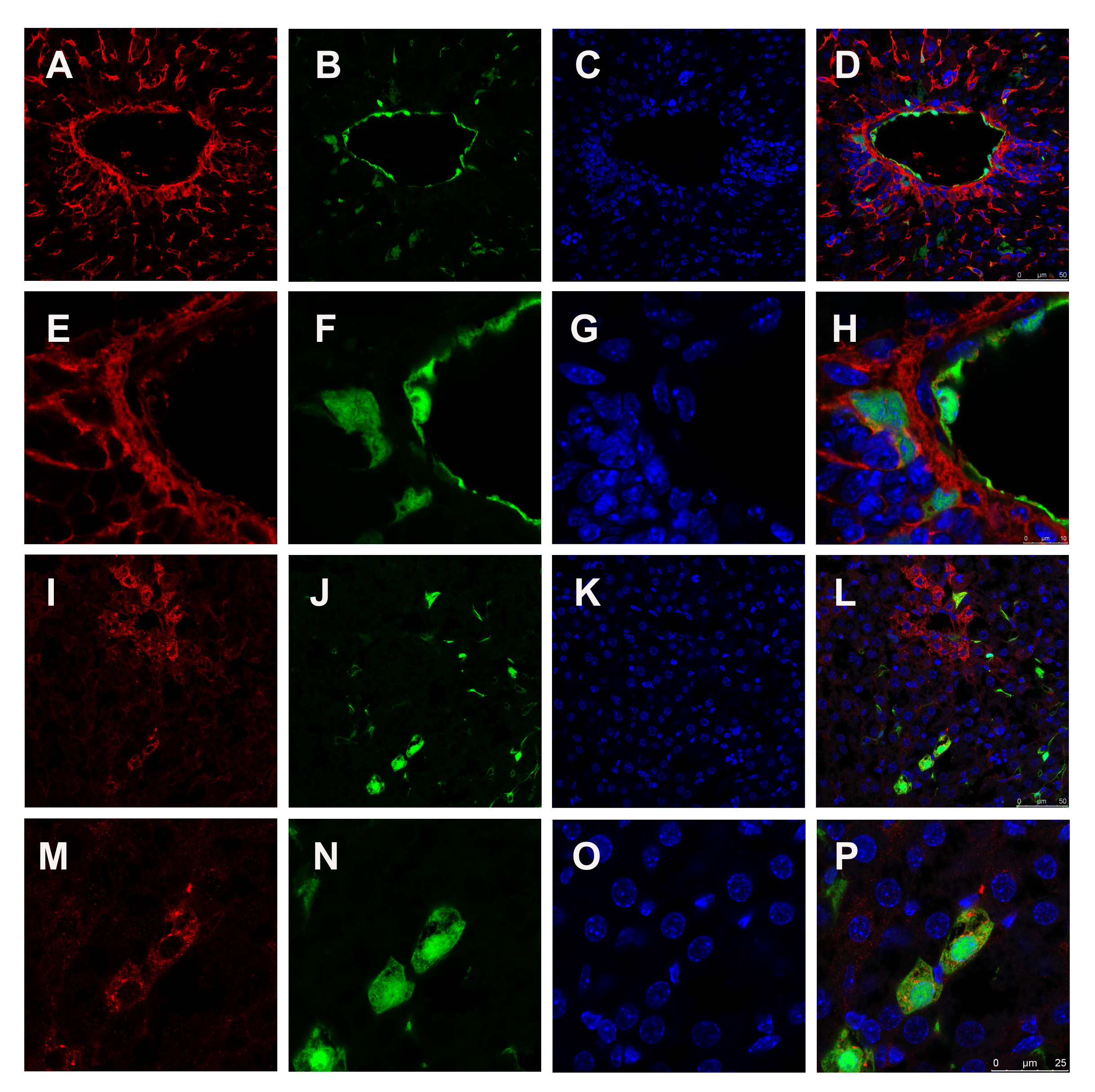
**

**Figure S5.** Integration of iPS cells in the liver of chimeric mice

The CD31 antibody-reactive cells (red, A and E) and AFP antibody-reactive cells (red, I and M) were detected specifically in the liver of chimeric mice from iPS cells of line 11.1. EGFP-positive cells (green, B, F, J and N) were the iPS cell-derived cells. Corresponding DAPI staining highlighting the nuclei is shown in panels C, G, K and O. The composite images are shown in panels D, H, L and P. The scale bars are 50 μm in A, B, C, D, I, J, K and L (low magnification), 25 μm in M, N, O (high magnification) and P, 10 μm in E, F, G and H (high magnification).
