# Supplementary material for: Germline-Competent Mouse-Induced Pluripotent Stem Cell Lines Generated on Human Fibroblasts without Exogenous Leukemia Inhibitory Factor
Source: PLoS One. 2009 Aug 21;4(8):e6724. doi: 10.1371/journal.pone.0006724 (PMC2725300; doi:10.1371/journal.pone.0006724)
Supplement: Table S1 — Primers for RT- PCR and genomic- PCR. (0.06 MB DOC) [file pone.0006724.s006.doc]

**Table S1. Primers for RT- PCR** and genomic- PCR

| **Gene Name** | **Forward/Reverse Sequence** |
| --- | --- |
| *ed-mMyc* | GCGTCCTGGGAAGGGAGATCCGGAGC |
|  | TTGAGGGGCATCGTCGCGGGAGGCTG |
| *ed-mOct4* | ATGGCATACTGTGGACCTCA |
|  | AGCAGCTTGGCAAACTGTTC |
| *ed-mSox2* | GGGAAATGGGAGGGGTGCAAAAGAGG |
|  | TTGCGTGAGTGTGGATGGGATTGGTG |
| *ed-mKlf4* | ACGATCGTGGCCCCGGAAAAGGACC |
|  | TGATTGTAGTGCTTTCTGGCTGGGCTCC |
| *ed-mGapdh* | AAAGTCAAGTTCTCCCACGAA |
|  | GGCACTTGCATGGAGTTTTCC |
| *tg-hOCT4* | CCCCAGGGCCCCATTTTGGTACC |
|  | TTATCGTCGACCACTGTGCTGCTG |
| *tg-hSOX2* | GGCACCCCTGGCATGGCTCTTGGCTC |
|  | TTATCGTCGACCACTGTGCTGCTG |
| *tg-hKLF4* | ACGATCGTGGCCCCGGAAAAGGACC |
|  | TTATCGTCGACCACTGTGCTGCTG |
| *tg-hMYC* | CAACCGAAAATGCACCAGCCCCAG |
|  | TTATCGTCGACCACTGTGCTGCTG |
| *ed-mNanog* | CTCATCAATGCCTGCAGTTTTTCA |
|  | CTCCTCAGGGCCCTTGTCAGC |
| *ed-Rex1* | ACGAGGTGAGTTTTCCGAAC |
|  | CCTCTGTCTTCTCTTGCTTC |
| *ed-Cdx2* | GACACCTTGGATCAGCTAAGCC |
|  | CCTCCAGCTCTATGACACACTG |
| *ed-Fgf5* | AAAGTCAATGGCTCCCACGAA |
|  | GGCACTTGCATGGAGTTTTCC |
| *ed-T* | GGTGGCTTGTTCCTGGTGC |
|  | GATGGTGGGCTGGCGTTAT |
| *ed-Sox7* | GACACCTTGGATCAGCTAAGCC |
|  | CCTCCAGCTCTATGACACACTG |
| *ed-Afp* | ATTCCTCCCAGTGCGTGAC |
|  | CAGCAGCCTGAGAGTCCAT |
| *ed-Ihh* | ACGTGCATTGCTCTGTCAAGT |
|  | CTGGAAAGCTCTCAGCCGGTT |
| *ed-Gsc* | TTCGGGAGGAGAAGGTGGA |
|  | CGGCGAGGCTTTTGAGGA |
| *mOct4*-Outside (Bisulfate seq) | GAGGATTGGAGGTGTAATGGTTGTT |
|  | CTACTAACCCATCACCCCCACCTA |
| *mOct4*-Inside (Bisulfate seq) | CAAGCTTTGGGTTGAAATATTGGGTTTATTT |
|  | CGGATCCCTAAAACCAAATATCCAACCATA |
