# Supplementary material for: Germline-Competent Mouse-Induced Pluripotent Stem Cell Lines Generated on Human Fibroblasts without Exogenous Leukemia Inhibitory Factor
Source: PLoS One. 2009 Aug 21;4(8):e6724. doi: 10.1371/journal.pone.0006724 (PMC2725300; doi:10.1371/journal.pone.0006724)
Supplement: Table S2 — Antibodies used in immunofluorescence staining. (0.03 MB DOC) [file pone.0006724.s007.doc]

**Table S2.** Antibodies used in immunofluorescence staining

| **Antibody** | **Company** | **Dilution** |
| --- | --- | --- |
| OCT4 | * | 1:500 |
| SOX2 | * | 1:500 |
| CD31 | DAKO | 1:50 |
| AFP | Chemicon | 1:100 |
| SOX17 | R&D | 1:50 |
| GATA4 | Santa Cruz | 1:50 |
| FOXA2 | R&D | 1:200 |
| NESTIN | Chemicon | 1:100 |
| OLIG2 | Chemicon | 1:100 |

*Antibodies were prepared in our lab.
